# Discrepant Results of Post-valve CT Analysis and Pulmonary Function Test in Patients Undergoing Bronchoscopic Lung Volume Reduction

**DOI:** 10.7759/cureus.93593

**Published:** 2025-09-30

**Authors:** Marianna Weaver, Prasanth Balasubramanian, Alanna Barrios-Ruiz, Ana Garza Salas, David Abia-Trujillo, Sebastian Fernandez-Bussy

**Affiliations:** 1 Pulmonary and Critical Care Medicine, Mayo Clinic, Jacksonville, USA; 2 Critical Care, Mayo Clinic, Jacksonville, USA; 3 Pulmonary Medicine, Mayo Clinic, Jacksonville, USA; 4 Division of Pulmonary, Allergy, and Sleep Medicine, Mayo Clinic, Jacksonville, USA

**Keywords:** bronchoscopic lung volume reduction, bronchoscopy, emphysema, endobronchial valves, total lung capacity

## Abstract

Introduction

Emphysema is a debilitating form of chronic obstructive pulmonary disease (COPD) that causes lung hyperinflation and air trapping, leading to reduced quality of life. Bronchoscopic lung volume reduction (BLVR) with endobronchial valves (EBVs) offers a minimally invasive treatment option by collapsing diseased lung segments to improve respiratory mechanics. While both CT and pulmonary function testing (PFT) are used to assess outcomes, it remains unclear whether post-procedural volume changes measured by CT correlate with functional improvements seen on PFT.

Materials and methods

We performed a single-center chart review of patients who underwent BLVR with EBV from January 2019 to December 2023 who had both pre-and post-procedure PFT and CT volume analysis performed. Data recorded included clinical and demographic characteristics, post-valve analysis, and PFT.

Results

A total of 14 patients were included in our study. A comparison of total lung volume change between post-BLVR CT analysis and PFT showed a -3% total volume change in post-BLVR CT analysis versus -6% total volume change in PFT. Post-BLVR CT analysis showed a median volume change of -247 mL versus PFT with -490 mL volume change. The volume change in PFT and post-BLVR CT analysis did not show a linear correlation (p = 0.315, R^2 ^= 0.101).

Conclusions

Our study found that post-BLVR pulmonary function testing showed greater volume reduction and functional improvement compared to CT analysis, suggesting a discrepancy between anatomical and physiological assessments. These findings indicate that CT may underestimate therapeutic response, and PFT may be a more reliable tool for evaluating clinical outcomes after EBV placement.

## Introduction

Emphysema, a subtype of chronic obstructive pulmonary disease (COPD), is a leading cause of morbidity and mortality worldwide. Despite adherence to guideline-based therapy, many patients remain symptomatic with poor quality of life. Bronchoscopic lung volume reduction (BLVR) using endobronchial valves (EBV) is a well-established minimally invasive procedure for patients with emphysema and severe air trapping with hyperinflation [1-3]. The procedure involves placement of one-way valves in targeted hyperinflated emphysematous lobes to allow passive lobar deflation, causing selective atelectasis of suboptimal lung parenchyma. Resultant reduction in lung volumes leads to measurable improvements in respiratory mechanics, diaphragm function, and ventilation-perfusion matching, leading to improved quality of life in patients.

Optimal patient selection is crucial to maximizing the benefits of EBV therapy and involves both pulmonary function testing (PFT) and CT analysis [4]. Studies have shown that CT analysis can predict suitable candidates with approximately 75% accuracy based on anatomical review of fissure integrity and lobar involvement [4-8]. However, CT analysis does not consider dynamic functional lung volumes. Post-procedure, CT imaging can be used to evaluate lung volume reduction, especially if the clinical response is suboptimal. Software analysis uses a quantitative model to identify inspiratory lung volumes but fails to consider functional lung volumes. Furthermore, variability in radiologist interpretation, patient inspiratory efforts, and imaging protocols can lead to confounded analysis [8]. Conversely, plethysmography on PFT is an already established quantitative marker of a patient’s functional lung volume. It remains unknown if these analyses correlate.

Despite the use of both CT imaging and PFT in the management of pre-BLVR, no studies to date have compared post-EBV lung volume measurements as measured by CT versus PFT. It remains unclear whether anatomical changes on CT correlate with functional improvement on PFT post-procedure. We hypothesized there would be significant discrepancies between anatomical and functional lung volumes, which could impact post-procedure clinical decision-making. This study aimed to compare lung volume changes as measured by CT analysis versus PFT, to provide guidance to clinicians on post-procedural assessment of patients.

## Materials and methods

We performed a single-center retrospective chart review of patients who underwent BLVR with EBV at Mayo Clinic Florida from January 2019 to December 2023. The study cohort included patients with severe COPD who underwent both pre- and post-procedure PFT and CT imaging. Collateral ventilation at the target lobe was assessed using CT imaging analyzed by the Chartis Pulmonary Assessment System and StratX (PulmonX Inc., Redwood City, CA). Patients who qualified for BLVR were treated with the Zephyr endobronchial valve (PulmonX Inc.). Post-procedural CT imaging was also processed using StratX to assess changes in lobar lung volumes. All CT imaging was performed at our institution. Chest CT scans were performed from the lung apices to the adrenal glands using Siemens Somatom AS/Edge/Force scanners with spiral acquisition (0.5-s rotation, 120 kVp, pitch 1.2). Images were reconstructed at 1.0-mm slice thickness with standard and sharp kernels, with multiplanar and MIP reconstructions generated as needed.

We collected and reviewed demographic characteristics (age, sex, BMI, smoking history), procedural details (number and location of valves placed, target lobe), and clinical outcomes, including PFT pre- and post-valve placement. Recorded PFT parameters included forced expiratory volume in one second (FEV1), total lung capacity (TLC), residual volume (RV), and forced vital capacity (FVC). All PFT tests were performed in the same lab according to the American Thoracic Society/European Respiratory Society (ATS/ERS) guidelines. CT volumetric analysis was performed using the described CT software. Only patients with both pre- and post-procedural PFT and CT data were included in the analysis.

This study was reviewed and deemed exempt by the institutional review board (#20-008945); all data were de-identified before analysis in compliance with institutional and federal privacy regulations.

Statistical analysis was performed using IBM SPSS Statistics software Version 28.0 (IBM Corp., Armonk, NY). Continuous variables were summarized using median and interquartile ranges (IQR), while categorical variables were reported as frequencies and percentages. Pre- and post-procedure lung volumes from PFT and CT analysis were compared using linear correlation analysis. P-values <0.05 were considered statistically significant.

## Results

A total of 14 patients qualified to participate in our study. The baseline characteristics of our patients are shown in Table 1. The median age of our patients was 68 years, with a median BMI of 25. All 14 identified themselves as white. All patients had a diagnosis of COPD, with 93% being former smokers. Other comorbidities included hypertension, diabetes mellitus, chronic kidney disease, obstructive sleep apnea, and alpha 1 antitrypsin deficiency. Oxygen was used by 71% of our patients before BLVR. Out of the 14 patients, 50% of the valves were placed in the left lower lobe, with 36% of the valves placed in the right upper lobe.

**Table 1:** Summary of baseline characteristics of patients included in study (N=14) BMI: body mass index; COPD: chronic obstructive pulmonary disease; RUL: right upper lobe; RML: right middle lobe; RLL: right lower lobe; LUL: left upper lobe; LLL: left lower lobe

Baseline characteristics	Values
Age, median (IQR)	68 (66-77)
Gender, n (%)	
Male	4 (29)
Female	10 (71)
Race, White, n (%)	14 (100)
BMI, median (IQR)	25 (23-28)
Smoking History, n (%)	13 (93)
Hypertension, n (%)	7 (50)
Diabetes mellitus, n (%)	2 (14)
Chronic kidney disease, n (%)	1 (7)
COPD, n (%)	14 (100)
Obstructive sleep apnea, n (%)	4 (29)
Alpha 1 antitrypsin deficiency, n (%)	2 (14)
Oxygen requirement, n (%)	10 (71)
Valve placement, n (%)	
LUL	1 (7)
LLL	7 (50)
RUL	5 (36)
RML	1 (7)
RLL	1 (7)
Multi-site	1 (7)

Pulmonary function testing pre- and post-valve placement is described in Table 2.

**Table 2:** Pre- and post-EBV placement pulmonary function parameters as measured by pulmonary function testing and CT analysis* ^*^Represented by the median (IQR) EBV: endobronchial valve; IQR: interquartile range; FEV1: functional expiratory volume; RV: residual volume; TLC: total lung capacity; DLCO: diffusing capacity of the lungs for carbon monoxide; CT: computed tomography

	Pre-EBV	Post-EBV	Volume change	Volume change (%)
PFT - FEV1 (ml)	665 (535-813)	695 (563-1000)	+100 (20 - 158)	16 (3 -32)
PFT - RV (ml)	5070 (4610-5780)	4220 (3880-4590)	-985 (-1388 - -513)	-20 (-23 - -7)
PFT - DLCO (ml/min*mmHg)	7.9 (4.8-9.3)	7.85 (5.4-9.2)	+0.5 (-0.3 - 1.7)	6 (-3 - 20)
PFT - TLC (ml)	7560 (7200-7990)	6740 (6650-7720)	-490 (-743 - -353)	-6 (-10 - -5)
CT - TLC (ml)	6704 (5493-7134)	6237 (5397-7082)	-247 (-439 - -39)	-3 (-6 – 0)

The median total functional lung capacity pre-valve placement was 7,560 mL with a post-valve median volume change of -6%. The amount of volume reduction for our patient population ranged from -743 ml to -353 ml. Forced expiratory volume in 1 second (FEV1) had an improvement of 16% after valve placement, with residual volume decreasing by 20%. Diffusion capacity had a 6% improvement after valve placement.

**Figure 1:**
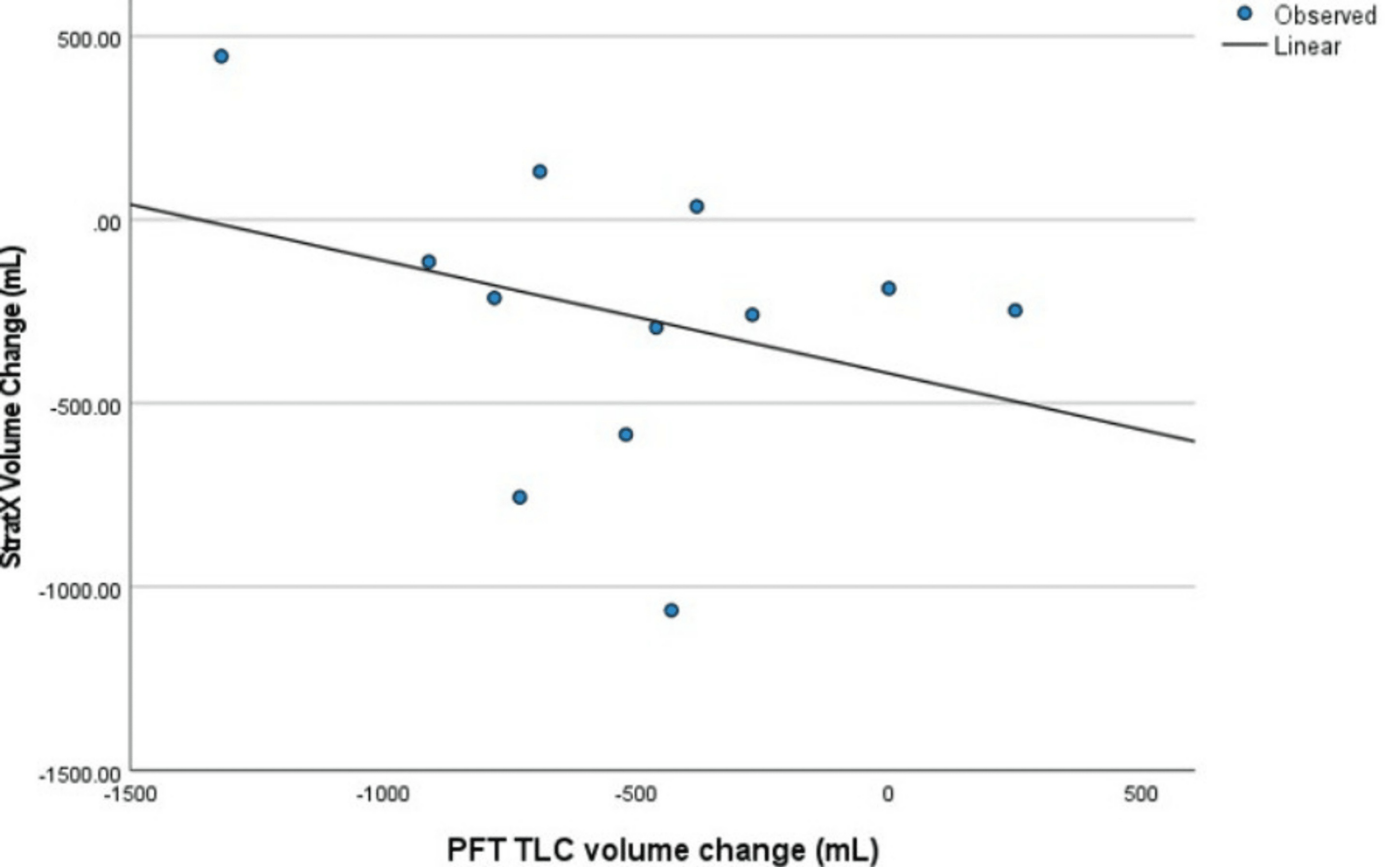
Lung volume change in PFT and CT analysis pre- and post-procedure showing no linear correlation Linear correlation: CT analysis volume = -418 + (-0.307 x TLC), R^2^ = 0.101, p = 0.315 PFT: pulmonary function test; CT: computed tomography; TLC: total lung capacity

A comparison of total lung volume change post-BLVR between PFT versus CT analysis (Table 2) shows a -3% total volume change in CT analysis versus -6% total volume change in PFT. CT analysis showed a median volume change of -247 mL versus pulmonary function testing with a -490 mL volume change. The volume change in PFT and CT analysis pre- and post-procedure did not show a linear correlation with each other, as shown in Figure 1 (p = 0.315, R^2^ = 0.101). Outlier values were identified and interpreted as variations in patient effort during testing, and did not impact the relationship of volume change in our linear correlation on further examination.

## Discussion

To date, there has been no published literature directly comparing PFT to CT analysis in assessing treatment response following BLVR with EBV. While CT analysis has been reported to have a 75% accuracy in predicting pre-valve placement through assessing fissure integrity and lobar hyperinflation, its use in post-valve placement has been poorly defined [4,5]. Despite the limitations posed by our small sample size, our findings in pulmonary function test results for pre- and post-EBV placement are consistent with those from other large-scale randomized controlled trials, such as LIBERATE and EMPROVE [9]. These trials showed significant improvement in FEV1 post-valve placement, representing functional improvement [3,10-12]. In our study, FEV1 improved by 16% or approximately 100 mL. Other smaller cohort studies showed similar findings in their PFTs [10,13-14]. This aligns with the LIBERATE trial, which reported an FEV1 increase of 17.2% at 12 months, and with EMPROVE demonstrating a 15.3% increase [11-12,15]. 

In addition to FEV1 improvement, our study showed a reduction in total lung capacity by 6% on PFT post-valve placement, aligning with other studies [10,14]. This is significant as the primary therapeutic aim of EBV placement. The VENT trial previously established a reduction in target lobe volume by >350 ml as being clinically significant [3]. In our cohort, median TLC reduction on PFT was -490 ml, while CT-based post-BLVR analysis estimated a median reduction of only -247 ml [16,17]. This discrepancy suggests that the post-BLVR CT analysis may underestimate true physiological volume changes, more accurately captured by PFT.

This underestimation of CT-based systems may be due to inter-center variability in CT acquisition protocols and analysis algorithms. Prior studies have described that CT analysis is susceptible to differences in ventilation criteria and reconstruction software, leading to inconsistent volume estimations [8,18]. Welling et al. hypothesized that high-resolution CT values may overestimate those estimated from PFT; however, this is not supported by our study [17]. In fact, our study suggests that PFT provides a more sensitive and consistent measure of lung volume change post-EBV placement, which suggests it as the preferred method for post-procedural evaluation. While PFT may represent functional change more accurately based on our results, we acknowledge the important role of CT imaging to assess valve placement and any complications, such as pneumothorax.

Limitations and future research directions

There are several limitations to our study. Firstly, the retrospective design of our study may introduce selection bias, while the small sample size restricts our statistical power. Not all patients underwent post-EBV CT analysis, resulting in an incomplete database for comparative evaluation. Another consideration is that a subset of our patients required later valve revisions, similar to other studies, and this was not factored into the results of our study [3,19]. 

Future studies should aim to prospectively quantify post-procedural lung volume changes using standardized CT protocols alongside PFT and investigate their correlation with long-term clinical outcomes, such as exercise capacity, dyspnea scores, and quality of life, as in the study by Brown et al., who followed patients for six months post-procedure, showing a reduction in their Borg scale score [13]. Given our small sample size, larger prospective studies should be performed to validate our findings. In addition, the incorporation of machine learning algorithms and artificial intelligence may create a standardized analysis that improves reliability.

## Conclusions

Our study highlights a lack of linear correlation between lung volume measurements obtained by CT analysis and PFT post-EBV placement. Given the variability and limitations of CT analysis, we recommend that clinicians use PFT data over CT-derived metrics when assessing patient response to EBV therapy.
